# Spontaneous Diaphragmatic Rupture after Coughing: Report of a Case and Review of the Literature

**DOI:** 10.1155/2019/1270195

**Published:** 2019-06-25

**Authors:** Hartwig Fenner, Elza Memeti, Stephanie Taha, Jürgen Fornaro, Andreas Scheiwiller, Jürg Metzger, Markus Gass

**Affiliations:** ^1^Department of Surgery, Cantonal Hospital of Lucerne, Lucerne, Switzerland; ^2^Department of Radiology, Cantonal Hospital of Lucerne, Lucerne, Switzerland

## Abstract

Spontaneous diaphragmatic rupture (SDR) is a very rare surgical emergency. A possible explanation for SDR might be a sudden increase of intra-abdominal pressure due to intense coughing, physical exercise, vomiting, or delivery. A 66-year-old male patient presented with recurrent coughing, dyspnoea, and intermittent fever. Although initial CT scan was inconspicuous, a follow-up CT scan revealed SDR of the left hemidiaphragm with herniation of the left colonic flexure, stomach, and parts of the greater omentum. Emergency laparotomy confirmed SDR. The ruptured anterior-lateral part of the diaphragm was closed, and additionally, a composite mesh was applied to reinforce the suture line. A right-sided hemicolectomy with primary anastomosis had to be performed. SDR is a rarity and can cause exceptional clinical features that may lead to inaccurate diagnosis and therapeutic delay. Therefore, of paramount importance, medical practitioners need to be aware of this important differential diagnosis for spontaneous dyspnoea or tachypnea.

## 1. Introduction

Rupture of the left diaphragm and herniation of intra-abdominal organs like the stomach, spleen, and colon are mostly caused by blunt or penetrating abdominal trauma. Spontaneous diaphragmatic rupture following coughing, delivery, or extensive exercises is very rare [1]. In an analogy to blunt abdominal trauma, it is hypothesized that the sudden rise of intra-abdominal pressure leads to the rupture of the weakest part, which is the diaphragm as in the presented case. Nevertheless, SDR is a surgical emergency and needs immediate surgical treatment and repair. Therefore, spontaneous chest pain and shortness of breath or tachypnea should lead to computer tomographic imaging to rule out differential diagnoses like pulmonary embolism, pneumonia, spontaneous pneumothorax, or SDR. We present a case of SDR with herniation of the stomach, liver, and left colonic flexure after severe coughing, and a review of the literature.

## 2. Case History/Clinical Examination

A 66-year-old male patient presented with back pain and infection-exacerbated COPD in our emergency department. Medical history revealed COPD Gold grade 1, hypertension, and alcohol abuse as relevant comorbidities. The reason for admission was severe prolonged coughing and dyspnea with fever up to 38 degree Celsius. The patient complained about constipation for three days and back pain as well as lower abdominal pain. Furthermore, he mentioned falling out of his bed during sleep the night before admission. He denied loss of appetite, nausea, and vomiting. Medical history revealed an arterial hypertension, an extensive alcohol abuse, and a 30-year history of cigarette smoking.

**Figure 1:**
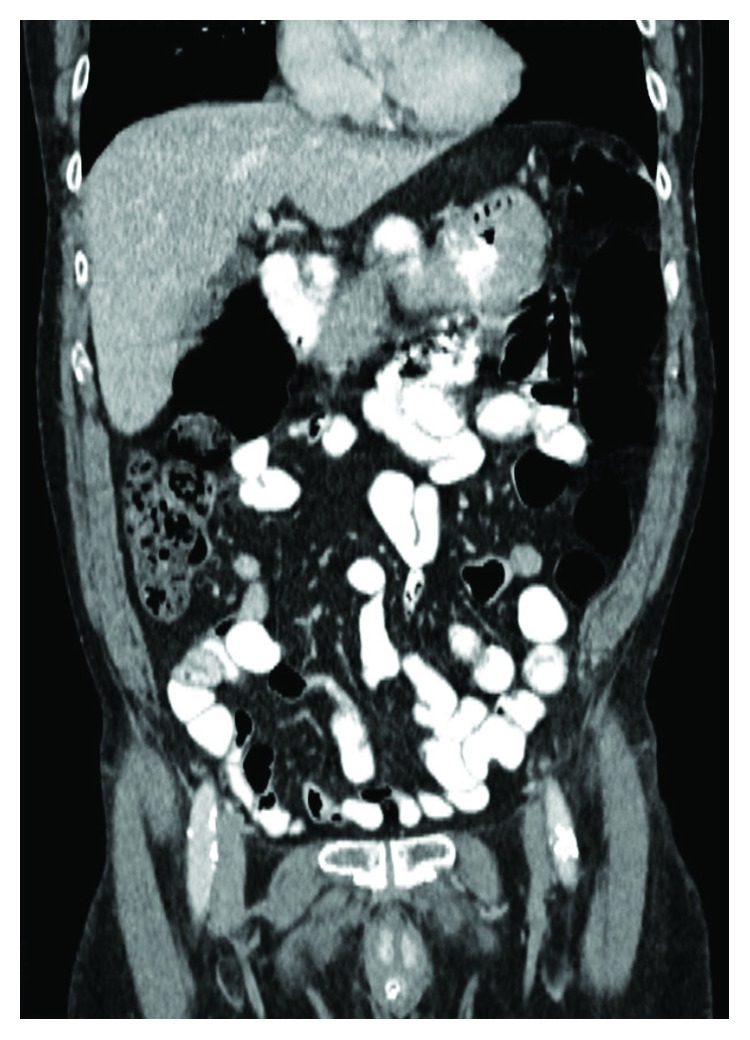
Coronary thoracoabdominal CT scan on admission without diaphragmatic hernia.

**Figure 2:**
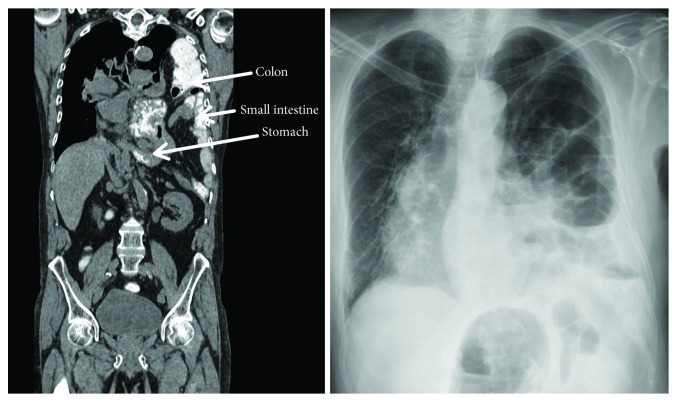
Coronary CT scan and chest X-ray 8 days after admission with large diaphragmatic hernia.

**Figure 3:**
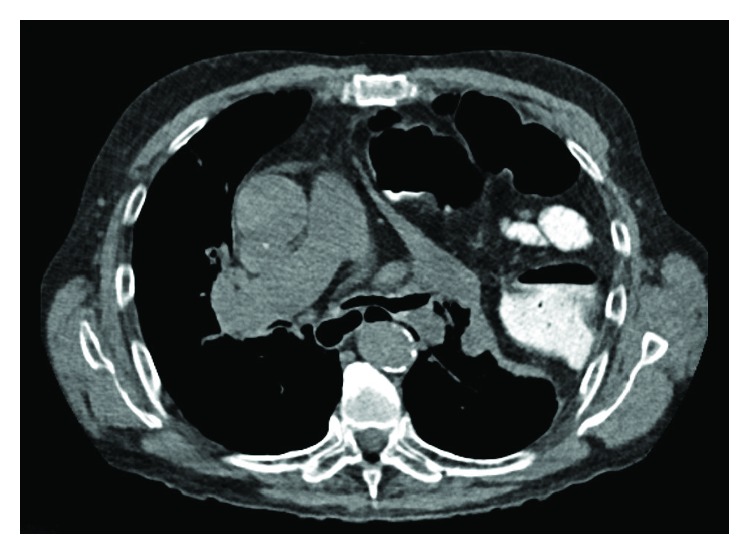
Axial CT scan demonstrating the diameter of the diaphragmatic hernia.

Clinical examination showed a bulging abdomen and diffuse pain on palpation. Normal bowel sounds were present. Further investigations showed a slightly elevated heart rate with 98 beats per minute, normal blood pressure, and oxygen saturation of 99% with 5 liters O_2_. Blood tests indicated no relevant systemic infection with leukocytosis of 11.27 × 10^9^/L and an elevated C-reactive protein (CRP) of 26 mg/L. Because of increasing CRP and leukocytes, antibiotic therapy was initiated. Initial CT scan demonstrated an intact diaphragm (Figure 1). The patient stayed in a bad general condition, leucocytes stayed stable, and CRP values rose to 60 mg/L. Eight days after admission, a severe respiratory insufficiency occurred and the patient had to be transferred to the intensive care ward. The CT scan was repeated and now showed a left diaphragmatic rupture with herniation of the stomach, spleen, and left colonic flexure (Figures 2 and 3).

## 3. Differential Diagnosis, Investigations, and Treatment

Subsequently, an explorative laparotomy was performed, the involved organs were reduced into the abdominal cavity, and blood and fluid were evacuated. Then, the diaphragm was primarily repaired with an absorbable running suture and additionally stabilized with a composite mesh. A thoracostomy tube was inserted to ensure reexpansion of the left lung and evacuate the pleural space. Obviously the right-sided colon was incarcerated, and a long serosal defect resulted. An extended right hemicolectomy had to be performed with an ileotransversostomy to restore intestinal continuity.

## 4. Outcome and Follow-Up

Unfortunately, two days after the initial operation, the patient developed signs of sepsis with tachycardia, hypotension, and abdominal peritonism. Revisional laparotomy showed a fascia dehiscence due to anastomotic leakage of the ileotransversostomy and intra-abdominal abscess. The composite mesh had to be removed, and an absorbable mesh was implanted. The anastomosis had to be resected, and a Hartmann procedure with terminal ileostomy was performed. The patient could finally be discharged after 52 days.

## 5. Discussion

Spontaneous diaphragmatic rupture is a very rare diagnosis in contrast to a rupture caused by blunt or penetrating abdominal trauma. About 30 cases are published in the literature to our knowledge. Most frequently, the ruptures are followed by a sudden increase of intra-abdominal pressure, e.g., vomiting, coughing, delivery, and heavy weight lifting, or generally spoken, they are mostly Valsalva-associated. Actually, these diaphragmatic ruptures cannot really be considered as “spontaneous” since independently if the increase of intra-abdominal pressure is caused by an external trauma or by the patient itself, always a massive increase of tension of the diaphragm is present. Most patients complain about chest pain, respiratory stress, and nausea with emesis. On physical examination, alarming vital signs can be present (tachycardia, hypotension, decrease of oxygen saturation, and fever) and the patient is restless and in distress. Due to herniation of the colon and small intestine, bowel sounds can be auscultated during chest examination whereas breathing sounds are decreased. With the results and suspicions of clinical examination, mostly the diagnosis is confirmed by chest X-ray or CT scan. Reduction of herniated organs and closure of the diaphragmatic defect is usually performed transabdominally. As in traumatic diaphragmatic rupture, a running suture as well as an interrupted suture is suitable. We prefer a strong absorbable running suture, and depending on the coexisting organ damage, we recommend in a noncontaminated abdominal cavity an augmentation with a composite mesh. In accordance to this, our patient has experienced two possible reasons for his diaphragmatic rupture. Before admission, he fell out of his bed, and on admission, he suffered from severe coughing. Although the initial CT scan and chest X-ray showed a regular anatomical situation of intra-abdominal and intrathoracical organs, maybe a clinically inapparent slight rupture became clinically symptomatic after the violent coughing episodes.

Barclay-Buchanan reported just two years ago a 69-year-old patient who presented at the emergency department with dyspnea after the Valsalva maneuver for chronic constipation. A CT scan showed a large anteromedial diaphragmatic rupture with herniation of the stomach, small intestine, and colon into the right hemithorax. The patient was admitted to the palliative care service for other reasons and died within 24 hours. A differential diagnosis in this patient could be incarceration of a preexisting undiagnosed diaphragmatic defect as a result of rising intra-abdominal pressure during bowel movements [1].

Hamaji et al. reported in 2013 another patient with SDR two days after her first uncomplicated vaginal delivery. A chest computed tomography revealed a left-sided diaphragmatic hernia with dislocation of the left colonic flexure and mesenteric and omental fat. A left oblique lateral thoracotomy was performed, and a 5 cm rupture of the diaphragm was diagnosed. After reduction of the herniated abdominal contents, the defect was repaired with two layers of nonabsorbable running stitches [2]. Very similar to our patient, Losanoff et al. reported some years ago a 36-year-old male patient with a viral illness and coughing [3]. Initial chest X-ray was inconspicuous, but after 24 hours, the repeated image showed herniation of the stomach. Intraoperatively, a 10 cm defect of the diaphragm was present, and analogously to our patient, the stomach, spleen, and left flexure of the colon were herniated. The defect was repaired with nonabsorbable figure-of-eight sutures, and in contrast to our patient, no visceral resection was necessary and the defect was not augmented by a mesh.

The literature review by Losanoff et al. found 28 detailed reports about SDR between 1956 and 2009; thus SDR is a very rare visceral emergency. Two-thirds of the patients (69%) suffered from a left-sided rupture and one patient from a bilateral defect [3]. The underlying origin for SDR was in this case series mostly coughing, followed by physical exercise or vaginal delivery.

Another rare origin of SDR is reported by Yang from Korea. As a result of static sport activity, a 29-year-old woman presented to the emergency department with worsening pain in the epigastric area after a Pilates session. On the CT scan, an air-fluid level of the stomach which was herniated in the left hemithorax was present. Left-sided thoracotomy revealed a 7 cm × 5 cm rupture of the left posterior medial diaphragm with a perforation of the stomach. 16 days after transthoracic repair of the diaphragm and stomach, the patient was discharged without any complications [4].

## 6. Conclusion

Spontaneous diaphragmatic rupture is an uncommon entity but a life-threatening condition. Mostly, the underlying mechanism for the tremendous increase of intra-abdominal pressure is not obvious and needs a low level of suspicion for timely accurate diagnosis and treatment. Although extremely rare, this possible differential diagnosis should be considered in patients with dyspnea, chest pain, and a history of an episode with Valsalva-like rise of intra-abdominal pressure and absent trauma. SDR is associated with high morbidity and mortality rates; therefore, early diagnosis is of paramount importance. Treatment options are exclusively surgical repair and exploration via a laparoscopic or open transabdominal or transthoracical approach and direct closure. An augmentation with a composite mesh can be decided on a case-by-case basis [5].
